# Job vacancy at the European Centre for Disease Prevention and Control (ECDC)

**DOI:** 10.2807/1560-7917.ES.2026.31.4.202601294

**Published:** 2026-01-29

**Authors:** Eurosurveillance editorial team

**Affiliations:** 1 European Centre for Disease Prevention and Control (ECDC), Stockholm, Sweden

The European Centre for Disease Prevention and Control (ECDC) invites applications for the following position:

**Data Scientist**
The application deadline expires on 20 February 2026.

For more information, please visit the ECDC recruitment platform: https://erecruitment.ecdc.europa.eu/?page=advertisement

